# Immunomodulatory Effects of Bacterial Toll-like Receptor Ligands on the Phenotype and Function of Milk Immune Cells in Dromedary Camel

**DOI:** 10.3390/biology12020276

**Published:** 2023-02-09

**Authors:** Jamal Hussen, Mayyadah Abdullah Alkuwayti, Baraa Falemban, Mohammed Ali Al-Sukruwah, Sameer M. Alhojaily, Naser Abdallah Al Humam, Salma Al Adwani

**Affiliations:** 1 Department of Microbiology, College of Veterinary Medicine, King Faisal University, Al-Ahsa 31982, Saudi Arabia; 2 Department of Biological Sciences, College of Science, King Faisal University, Al Ahsa 31982, Saudi Arabia; 3 Department of Biomedical Sciences, College of Veterinary Medicine, King Faisal University, Al-Ahsa 31982, Saudi Arabia; 4 Agricultural and Veterinary Training and Research Station, King Faisal University, Al-Ahsa 31982, Saudi Arabia; 5 Department of Animal & Veterinary Sciences, Sultan Qaboos University, Muscat 123, Oman

**Keywords:** Toll-like receptor ligands, dromedary camel, milk cells, innate immunity, flow cytometry, phagocytosis

## Abstract

**Simple Summary:**

Mastitis is one of the most challenging diseases of dairy animals, with a high impact on animal production and welfare. The development of mastitis vaccines requires deep understanding of host–pathogen interaction mechanisms in the mammary gland. In the present study, we **i**nvestigated the immunomodulatory effect of selected Toll-like receptor (TLR) ligands, representing gram-positive and gram-negative bacterial mastitis pathogens, on the phenotype and function of milk immune cells in a whole milk stimulation assay. The analysis of stimulation-induced shape change, change in the expression of cell surface markers, phagocytosis, apoptosis, ROS production, and NETosis revealed selective modulating effects of the TLR ligands LPS and Pam3CSK4 on camel milk immune cells. These results may have implications for the use of synthetic TLR agonists as immunomodulatory adjuvants of the immune response to intra-mammary vaccines against mastitis pathogens.

**Abstract:**

(1) Toll-like receptors (TLR) are a family of pattern recognition receptors that sense distinct molecular patterns of microbial origin. Although the immune cell composition of camel milk has been recently described, host–pathogen interaction studies in the camel mammary gland are still scarce. The present study aimed to use a whole milk stimulation assay for investigating the modulatory effect of selected Toll-like receptor (TLR) ligands on the phenotype and function of milk immune cells. (2) Methods—camel milk samples (*n* = 7) were stimulated in vitro with the TLR4 ligand LPS or the TLR2/1 ligand Pam3CSK4, and separated milk cells were evaluated for stimulation-induced shape change, the expression of cell surface markers, phagocytosis, apoptosis, ROS production, and NETosis. Stimulation with PMA was used as a control stimulation. (3) Results—all stimulants induced shape change in milk cells, change in the expression of several cell markers, and increased cell apoptosis and NETosis. In addition, stimulation with Pam3CSK4 and PMA was associated with enhanced ROS production, while only PMA stimulation resulted in enhanced bacterial phagocytosis by milk immune cells. (4) Conclusions—our data indicates selective modulating effects of the TLR ligands LPS and Pam3CSK4 on camel milk phagocytes. These results may have implications for the use of synthetic TLR agonists as immunomodulatory adjuvants of the immune response to intra-mammary vaccines against mastitis pathogens.

## 1. Introduction

Mastitis is one of the most common and costly diseases in camels [1]. Recent studies report a similar pathogen spectrum for camel and cattle mastitis, which mainly includes the gram-positive bacteria *Staphylococcus* and *Streptococcus* and the gram-negative coliform bacteria (*E. coli, Klebsiella* spp., and *Enterobacter* spp.) [2]. However, the host–pathogen interaction mechanisms in the camel mammary gland have not been investigated. A clear understanding of these mechanisms is essential for the development of preventative and therapeutic strategies.

The mammary gland immune system consists of several humoral and cellular factors that contribute to the elimination of mastitis pathogens [3,4,5,6]. An effective immune response in the mammary gland depends on the early sensing of pathogens, triggering innate effector mechanisms, and the subsequent activation of the adaptive immune response [7,8,9,10,11,12].

The innate immune response to infection is triggered upon the recognition of pathogen structures called the pathogen-associated molecular patterns (PAMPs) and danger molecules called damage-associated molecular patterns (DAMPs) via pattern recognition receptors (PRRs) expressed by host cells [7,13]. Toll-like receptors (TLR) are one of the PRR members expressed on and in innate cells, such as macrophages, dendritic cells, monocytes, neutrophils, and epithelial cells [8,9,10,11,12,14,15,16,17].

For gram-negative and gram-positive bacterial pathogens, different PAMPs have been identified. Lipopolysaccharide (LPS) is a cell wall endotoxin of gram-negative bacteria that contributes to the local and systemic inflammatory response associated with acute coliform mastitis in dairy animals [18,19]. The innate recognition of LPS is mediated by the interaction between TLR4, the cluster of differentiation (CD) 14, serum LPS-binding protein, and myeloid differentiation factor-2 (MD-2), leading to the upregulation of several inflammatory cytokines [20,21,22]. Additionally, Pam3CSK4 is a TLR1/2 ligand representing a PAMP that mimics the innate response to infections with gram-positive bacteria, such as the *Streptococcus* and *Staphylococcus* species [23,24].

The ©mmune cell composition of camel milk was recently described [25]. Camel milk leukocytes are classified based on their differential expressionof several myeloid cell markers, including CD172a, MHCII, and CD14, into a dominant granulocyte population (CD172a+/CD14low/SSC^high^) followed by smaller populations of macrophages (CD172a+/CD14l^high^/SSC^high^) and lymphocytes (CD172a^−^/CD14^−^/SSC^low^) [25].

Effector cellular mechanisms against bacterial infections of the mammary gland mainly include early phagocytosis and the subsequent killing of pathogens through the production of reactive oxygen and nitrogen species by milk macrophages and neutrophils, degranulation, and the release of the antimicrobial peptides stored in the intracellular granules of neutrophil and the formation of neutrophils extracellular traps [26,27,28,29].

For several veterinary species, especially the dairy cow, different innate immune responses are observed after intra-mammary infections with gram-positive and gram-negative bacteria [30,31,32,33]. Experimental infection of the bovine mammary gland with live *S. aureus* or *E. coli* bacteria or the intra-mammary activation of TLR2 or TLR4 through heat-killed *S. aureus* or *E. coli* bacteria, respectively, results in the production of different cytokine patterns. Studies linked the selectively reduced production of the inflammatory cytokines TNF-α and IL-8 in *S. aureus*-infected quarters compared to the *E. coli*-infected quarters to the ability of the pathogens to induce acute or chronic mastitis, respectively [30,34]. Given the lack of knowledge regarding the pathogen species-specific response in the mammary gland of dromedary camels, the objective of the present study was to analyze the impact of the TLR 4 ligand LPS and the TLR 2/1 ligand Pam3CSK4, which are representative PAMPs for gram-positive and gram-negative bacteria, respectively, on the phenotype and function of immune cells in camel milk.

## 2. Materials and Methods

### 2.1. Animals and Sampling

Milk samples were collected during the first month of lactation from seven dromedary she-camels selected from a camel herd reared on a private camel farm located in the Al Hofuf city in the Eastern Province of Saudi Arabia. To exclude the impact of animal breed and age on the analysis, the selected animals were all of Al-Majaheem breed and were between 8 and 10 years old. Animals with clinical or subclinical mastitis were identified and excluded from the analysis based on a clinical examination of the mammary gland (swelling and pain in the infected quarters), abnormal physical properties of the milk (changed color and consistency), and the results of the California mastitis test (CMT). In addition, camels with a fever or clinical signs of any other infectious disease, such as respiratory infections, diarrhea, and joint infections, were excluded from the sampling. After discarding the first milk jets and cleaning and disinfecting the teat ends, the milk samples (150 mL) were collected into sterile glass bottles for whole milk activation [35,36]. The CMT results were evaluated according to the Scandinavian scoring system [35]. Milk samples with a score ≥ 3 were excluded from the analysis. The collected milk samples were kept cooled in a cooler until they were transported to the lab (within 4 h after collection).

### 2.2. In Vitro Whole Milk Stimulation with TLR Ligands

Whole milk stimulation was performed as described previously for in vitro stimulation of human milk samples with some modifications [37]. The milk samples (*n* = 7 animals) were stimulated with the TLR4-ligand LPS, the TLR2/1-ligand Pam3CSK4 (Palmitoyl-Cys((RS)-2,3-di(palmitoyloxy)-propyl)-Ser-Lys-Lys-Lys-Lys-OH), the protein kinase C activator phorbol 12-myristate 13-acetate (PMA), or only RPMI medium (Sigma-Aldrich, St. Louis, MO, USA) without stimulants. Here, Pam3CSK4 and ultrapure LPS from *E. coli* serotype 0111:B4 were obtained from Invivogen (Toulouse, France), diluted in endotoxin-free water to a stock concentration of 1 mg/mL, and stored at −20 °C. The PMA was obtained from Calbiochem (Darmstadt, Germany), diluted in dimethylsulfoxide (DMSO; Merck Millipore, Darmstadt, Germany) to a stock concentration of 5 mg/mL, and stored at −20 °C. The stimulants LPS and Pam3CS4K were selected as pathogen-associated molecular patterns that are representative of gram-negative and gram-positive bacteria, respectively. Here, PMA, which is a mitogen that is commonly used as an immune cell stimulator, was used as a positive control. For the in vitro stimulation, 20 mL of pooled milk samples (prepared from quarter milk samples for each animal) were incubated in conical 50 mL sterile polypropylene conical tubes (Merck Millipore, Darmstadt, Germany) with an equal volume of RPMI-1640 cell culture medium (supplemented with 100 U/mL penicillin and 50 μg/mL streptomycin). The tubes contained either 1 µg/mL ultrapure LPS, 1 µg/mL Pam3CSK4, 500 ng/mL PMA, or were left without any stimulant (medium control). The tubes were incubated for 18 h at 37 °C and 5% CO_2_. During the incubation, the tubes were inverted several times.

### 2.3. Separation of Milk Cells

For cell separation, the stimulated milk samples were centrifuged for 20 min (1000× *g*, 4 °C, without brake). After centrifugation, the fat layer was removed using a spatula, and the milk supernatant was discarded. The remaining cell pellet at the bottom of the tube was then washed twice with 30 mL of cold PBS (600× *g* and 4 °C for 10 min). Finally, the milk cell pellet was suspended in the flow cytometry buffer (PBS supplemented with 5 g/L bovine serum albumin and 100 mg /L NaN3) or in RPMI-1640 cell culture medium at a concentration of 1 × 10^6^ cells/mL. Cell vitality was measured after labeling the cells with propidium iodide (PI; 2 µg/mL, Calbiochem, Germany), and dead/necrotic cells were identified as PI + cells.

### 2.4. Labeling Milk Cells with Monoclonal Antibodies

The separated milk cells (1 × 10^5^ cells in 100 µL per well) were incubated with unlabeled primary monoclonal antibodies [38,39,40,41,42] in the following combinations: mouse IgG1 anti-CD14 (clone Tuk4) with mouse IgG2a anti-MHCII (clone TH81A5) and mouse IgG1 anti-CD163 (clone LND68A) with mouse IgG2a anti-CD44 (clone LT41A). All the monoclonal antibodies were from Kingfisher (Kingfisher Biotech. Inc., St. Paul, MN 55114, USA). After a 15 min incubation on ice at 4 °C, the unbound antibodies were removed by washing the cells with flow cytometry buffer (centrifugation for 3 min at 300× *g* and 4 °C). To detect the primary antibodies, secondary anti-mouse IgG1 or IgG2a antibodies labeled with different fluorochromes (Thermo Fisher Scientific, Waltham, MA, USA) were added to the wells in a second staining step. The plate was then incubated for another 15 min at 4 °C in the dark. For the CD18 staining, the cells were directly incubated with a mouse anti-CD18 antibody (BD Biosciences, San Jose, CA, USA) labeled with fluorescein isothiocyanate (FITC). Subsequently, the cells were washed twice with flow cytometry buffer (centrifugation for 3 min at 300× *g* and 4 °C), resuspended in 150 µL of buffer, and finally analyzed by flow cytometry (Accuri C6 flow cytometer, BD Biosciences). For all the investigations, staining only with antibody isotype controls (Mouse IgG1, IgG2, IgM isotype controls; BD Biosciences, USA) was included.

### 2.5. Bacterial Phagocytosis Assay

The analysis of the phagocytosis function of milk phagocytes was performed after incubating the milk cells with heat-killed *S. aureus* bacteria (Pansorbin, Merck, Nottingham, UK) labeled with a fluorescein isothiocyanate labeling kit (Sigma-Aldrich, St. Louis, MO, USA) as recommended by the manufacturer [43]. The separated milk cells (1 × 10^5^ in 100 µL RPMI cell culture medium) were incubated (37 °C and 5% CO_2_) with *S. aureus*-FITC (30 bacteria per cell) for 45 min. Finally, to remove the unbound bacteria, the cells were washed (300× *g* for 3 min) with RPMI medium, resuspended in 150 μL of PBS, and analyzed on the Accuri C6 flow cytometer.

### 2.6. Cell Apoptosis Assay

The mitochondrial membrane potential (MMP) probe JC-1 (5,5′,6,6′-tetrachloro-1,1′,3,3′-tetraethylbenzimidazolcarbocyanine iodide; Thermo Fisher Scientific, Waltham, MA, USA) was used for the apoptosis measurement in the milk cells, as previously described [44,45,46,47]. The separated milk cells (1 × 10^5^ cells in 100 µL of RPMI cell culture medium) were incubated with 100 µL of JC-1 solution (2 μmol/L final concentration) in a 96-well microtiter plate for 20 min at 37 °C and 5% CO_2_. After incubation, the cells were washed twice with PBS, suspended in 150 μL of PBS, and acquired on the flow cytometer (BD Accuri C6 flow cytometer). Milk cells containing JC-1 monomers (apoptotic cells) were differentiated from cells with JC-1 aggregates (viable cells) based on their emission in the green (FL1) and orange fluorescence channels (FL2), respectively, upon excitation at 488 nm.

### 2.7. Flow Cytometric Analysis of Neutrophil Extracellular Trap (NET) Formation

The SYTOX^®^ Green nucleic acid dye (Thermo Fisher Scientific, Waltham, MA, USA) was used for the analysis of NET-formation in milk cells as previously described [48]. Milk cell labeling was performed in a round-bottomed 96-well microtiter plate by incubating 100 µL of the cell suspension (1 × 10^5^ cells per well) with 50 µL of the DNA-sensitive dye SYTOX^®^ Green. After incubation for 15 min at room temperature, cell acquisition was carried out using the Accuri C6 flow cytometer (BD Biosciences).

### 2.8. Statistical Analyses

The software program Prism (GraphPad software version 5, GraphPad Software, San Diego, CA, USA) was used for statistical analysis of the results. A normal distribution of the data was assessed using the Shapiro–Wilk test. The comparison between the cells from unstimulated milk samples and samples stimulated with LPS, Pam3C4K, or PMA was performed using an ANOVA (one-factor analysis of variance) test in combination with Bonferroni’s multiple comparison test. The statistical results are presented graphically for each parameter as the mean ± SEM (standard error of the mean). The differences were considered significant if the *p*-value was less than 0.05.

## 3. Results

### 3.1. Stimulation-Induced Shape Change in Milk Cells

**Figure 1:**
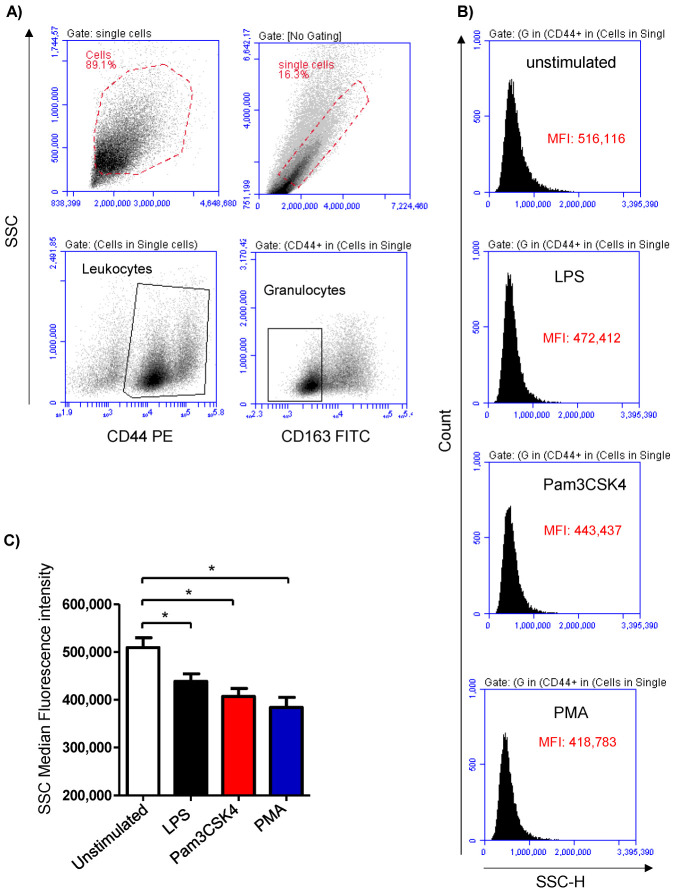
Flow cytometric analysis of stimulation-induced shape-change in milk granulocytes. Cells were separated from stimulated and non-stimulated milk samples, stained with antibodies for CD163 and CD44, and analyzed using flow cytometry. (**A**) Granulocytes were identified as SSC^high^CD44^+^CD163^-^ cells within the SSC^high^ cells. (**B**) Histograms showing the mean SSC values for unstimulated and stimulated cells. (**C**) Mean fluorescence intensity (MFI) was calculated and presented for stimulated and unstimulated cells; * indicates statistically significant differences between the groups (*n* = 7 camels; *p* values less than 0.05).

Cell stimulation was analyzed based on the induced change in the granulocyte’s side scatter (SSC) signal, which is indicative of cell granularity (Figure 1A,B). Stimulation of the milk samples with either LPS (438,699 ± 15,805 MFI), Pam3CS4K (407,113 ± 16,688 MFI), or PMA (384,031 ± 21,135 MFI) induced a significant (*p* < 0.05) decrease in the SSC MFI compared to the SSC signals of the cells from the unstimulated (509,414 ± 20,864 MFI) milk samples (Figure 1C).

### 3.2. Impact of TLR-Ligands on Milk Leukocyte Apoptosis

**Figure 2:**
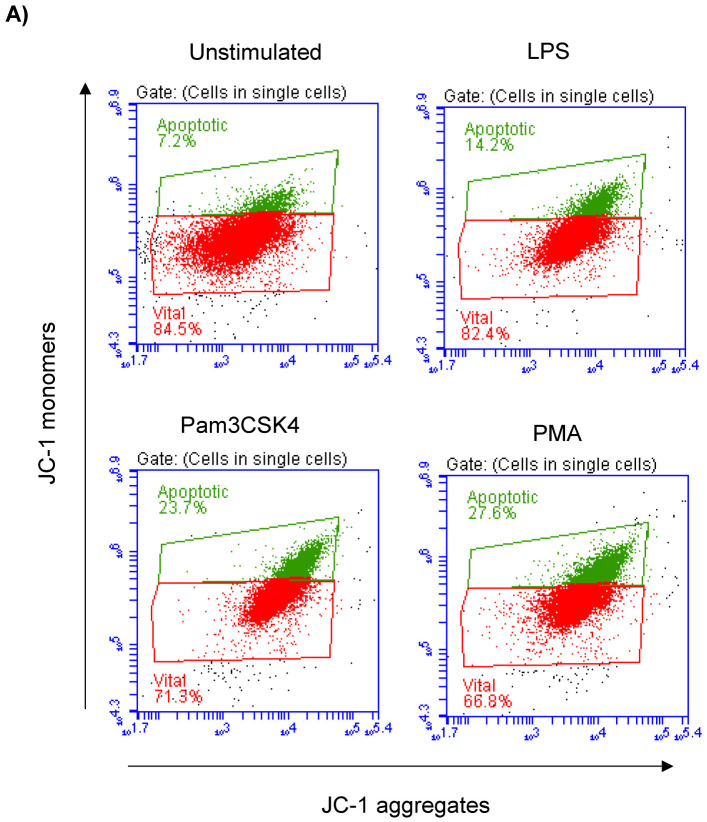

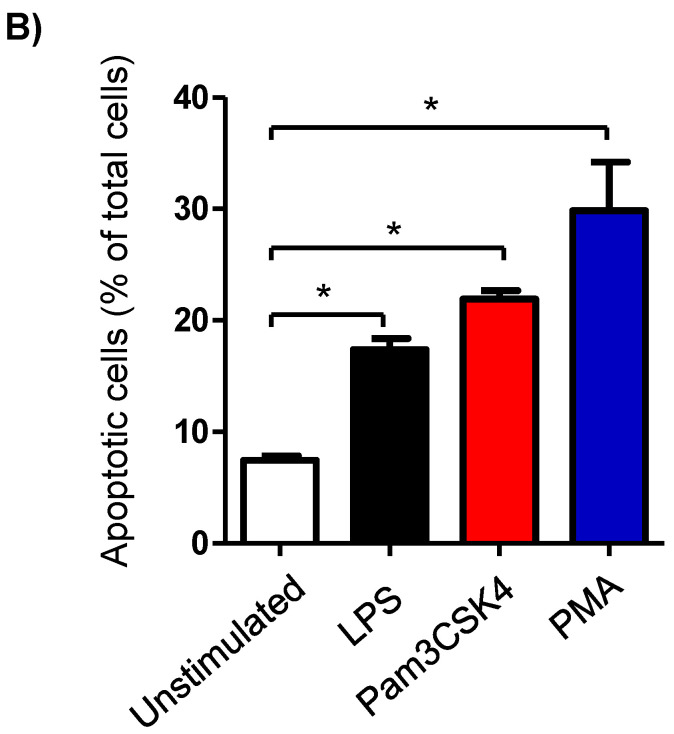
Flow cytometric analysis of apoptosis in milk cells. Cells were separated from unstimulated and stimulated milk samples, stained with JC-1, and analyzed by flow cytometry. (**A**) Apoptotic cells (with JC-1 monomers) were differentiated from viable cells (with JC-1 aggregates) based on their emission in the green (FL1) and orange fluorescence channels (FL2), respectively, upon excitation at 488 nm. (**B**) Data were calculated for stimulated and unstimulated milk cells and presented graphically. * indicates statistically significant differences between the groups (*n* = 7 camels; *p* values less than 0.05).

Milk cell apoptosis was evaluated using flow cytometry and the mitochondrial membrane potential (MMP) probe JC-1 (Figure 2A). The percentage of apoptotic cells in the unstimulated milk samples was 7.4 ± 0.3% of the total cells. Stimulation of the milk samples with LPS (17.4 ± 0.9% of total cells), Pam3CS4K (21.9 ± 0.7% of total cells), or PMA (29.9 ± 4.3% of total cells) resulted in a significant (*p* < 0.05) increase in the percentage of apoptotic cells. The strongest pro-apoptotic effect was, however, induced by stimulating the cells with PMA. The difference was only significant between the PMA-stimulated and LPS-stimulated cells (Figure 2B).

### 3.3. Modulatory Effects of TLR-Ligands on the Expression Level of Cell Surface Antigens on Milk Cells

**Figure 3:**
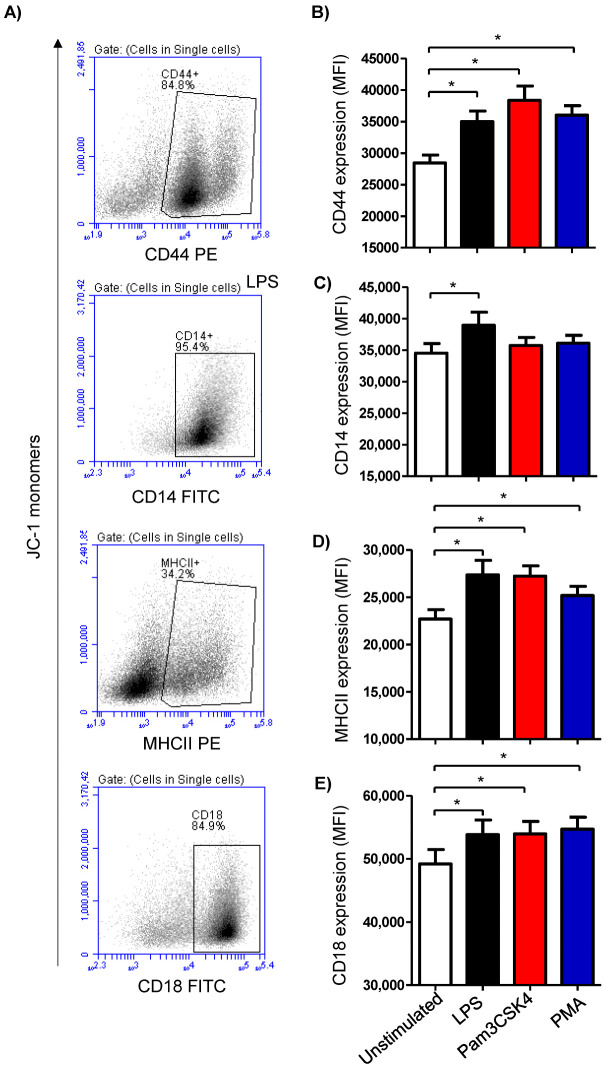
Analysis of the expression of the cell marker antigens CD44, CD14, MHCII, and CD18 on milk cells. Cells were separated from stimulated and non-stimulated milk samples, stained with monoclonal antibodies, and analyzed using flow cytometry. (**A**) Representative density plots (SSC-H against specific staining with the corresponding antibody) showing the expression density of the cell surface antigens after setting a gate on single cells. (**B**) Mean fluorescence intensity (MFI) of CD44 (**B**), CD14 (**C**), MHCII (**D**), and CD18 (**E**) on milk cells (*n* = 7 camels). * indicates statistically significant differences between the groups (*n* = 7 camels; *p* values less than 0.05).

The expression density of the cell surface molecules CD14, MHCII, CD44, and CD18 was analyzed on cells from the stimulated and unstimulated milk samples (Figure 3A). Stimulating the milk samples with either LPS (27,365 ± 1519 MFI; mean fluorescence intensity), Pam3CS4K (27,217 ± 1099 MFI), or PMA (25,170 ± 973 MFI) induced a significant (*p* < 0.05) upregulation of the monocytic activation marker MHCII on the milk cells in comparison to the cells from the unstimulated samples (22,694 ± 998) (Figure 3B). The expression density of CD14 was, however, only increased (*p* < 0.05) after stimulation with LPS (38,933 ± 2075 versus 34,524 ± 1518 for unstimulated cells) (Figure 3B). For the cell adhesion molecules CD44 and CD18, all the stimuli induced a significant (*p* < 0.05) rise in their expression densities in the milk cells (Figure 3B).

### 3.4. Impact of TLR-Stimulation on the Phagocytosis Activity of Milk Cells

**Figure 4:**
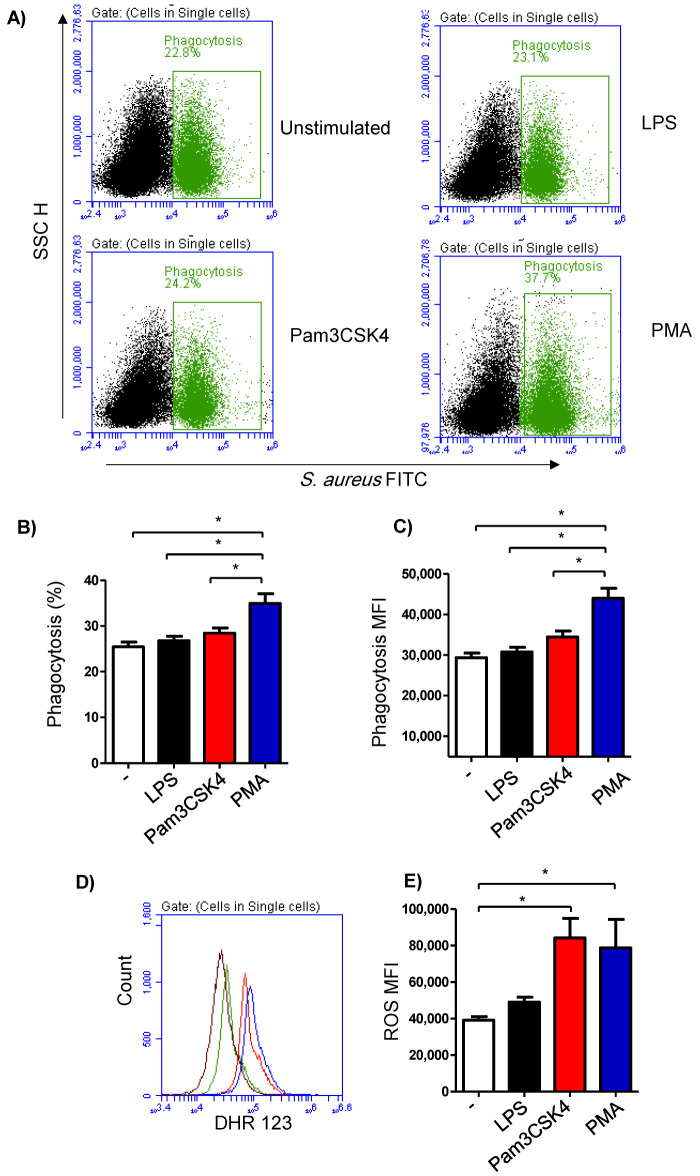
Flow cytometric analysis of phagocytosis and ROS production by milk cells. (**A**) Cells were separated from unstimulated and stimulated milk samples, incubated with FITC-conjugated *S. aureus* bacteria, and analyzed by flow cytometry. Phagocytosis-positive cells were identified based on their enhanced green fluorescence in FL-1. The percentage of phagocytosis-positive cells (**B**) as well as their main fluorescence intensity (**C**) were calculated for stimulated and unstimulated milk cells and presented graphically. (**D**) ROS production was analyzed after staining the cells with DHR-123 by measuring the mean green fluorescence intensity using flow cytometry. (**E**) ROS production in unstimulated and stimulated cells was measured and presented graphically (*n* = 7 camels). * indicates statistically significant differences between the groups (*n* = 7 camels; *p* values less than 0.05).

The phagocytosis activity of the milk cells was analyzed by flow cytometry using FITC-labelled *S. aureus* bacteria (Figure 4A). The fraction of phagocytosing cells was 25.4 ± 4.3% of the total cells in the unstimulated milk samples (Figure 4B). In milk samples stimulated with PMA (36.9 ± 2.4% of total cells), there was a significantly (*p* < 0.05) higher percentage of phagocytosing cells in comparison to the samples stimulated with LPS (26.8 ± 1.0% of total cells) or Pam3CS4K (28.4 ± 1.1% of total cells). Stimulation with either LPS or Pam3CS4K did not induce (*p* > 0.05) a significant change in the percentage of phagocytosing cells within the total milk cells (Figure 4B). Similarly, the phagocytosis index (mean fluorescence intensity), which represents the number of bacteria phagocytosed by each cell, was highest for the cells stimulated with PMA (*p* < 0.05). In addition, stimulation with Pam3CS4K increased (*p* < 0.05) the phagocytosis index in comparison to the unstimulated milk samples (Figure 4C).

### 3.5. Impact of TLR-Stimulation on the ROS-Production Activity of Milk Cells

Stimulation-induced ROS production was measured using the fluorescent dye DHR 123 (Figure 4D). The amount of ROS in the cells from the unstimulated milk samples was 39,105 ± 2047 MFI (median fluorescence intensity). Although a slight increase in the ROS signal was observed in the cells from the LPS-stimulated milk samples (49,015 ± 2660 MFI), the change was not statistically significant (*p* > 0.05) in comparison to the ROS signal in unstimulated samples. Stimulation with either Pam3CS4K (84,234 ± 10,614 MFI) or PMA (78,783 ± 15,535 MFI) resulted in a significant rise in the produced ROS from the milk cells (Figure 4E).

### 3.6. Formation of NETs in Stimulated Milk Cells

**Figure 5:**
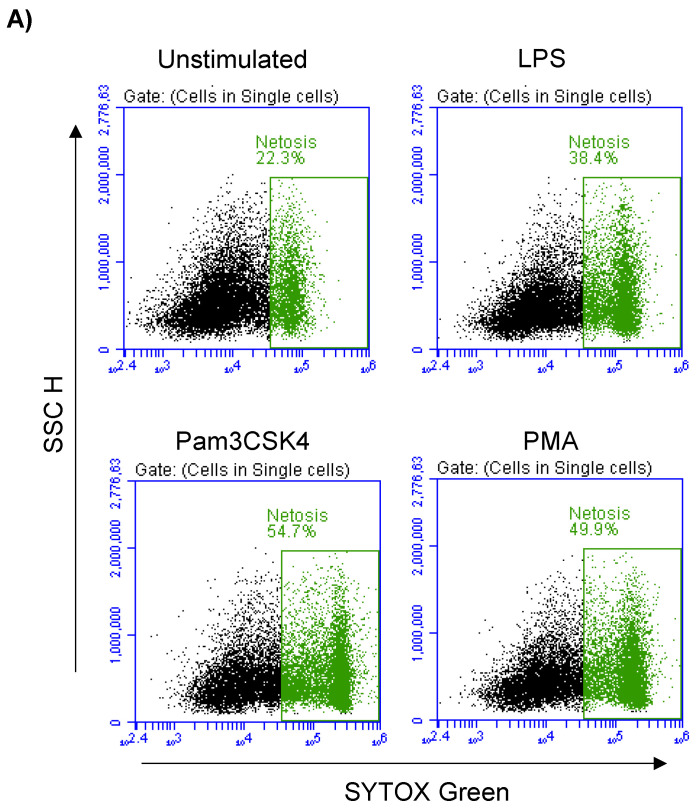

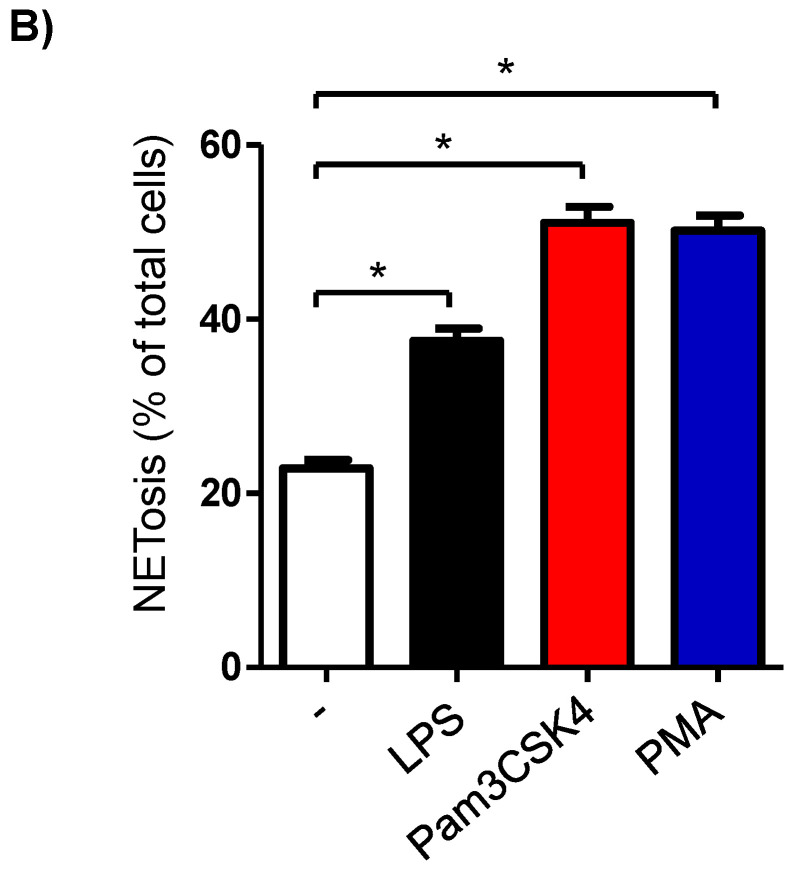
Flow cytometric analysis of NET-formation (NETosis) in milk cells. Cells were separated from unstimulated and stimulated milk samples, stained with Cytox Green, and analyzed by flow cytometry. (**A**) Here, NET-formation was identified based on the enhanced green fluorescence in FL-1. (**B**) The percentage of cells with NET-formation was calculated for stimulated and unstimulated milk cells and presented graphically (*n* = 7 camels). * indicates statistically significant differences between the groups (*n* = 7 camels; *p* values less than 0.05).

In the unstimulated milk samples, the percentage of NETosis-positive milk cells showing positive staining by the SYTOX^®^ Green dye (Figure 5A) was 22.7 ± 0.9% of total cells. This fraction was significantly (*p* < 0.05) increased after stimulation with LPS (37.5 ± 1.4% of total cells), Pam3CSK4 (51.1 ± 1.8% of total cells), and PMA (50.2 ± 1.8% of total cells). The stimulation-induced NET-formation was higher (*p* < 0.05) for the cells stimulated with Pam3CSK4 or PMA in comparison with the cells stimulated with LPS (Figure 5B).

## 4. Discussion

Mammary gland infections are among the most common diseases in camels, with an impact on animal production and welfare [49]. Although recent studies report a similar prevalence and mastitis-inducing pathogen spectrum for camels and cattle, it is still unknown whether the immune response of the mammary gland to those pathogens is the same in the two species [50,51,52]. Understanding the immune mechanisms in the camel mammary gland would pave the way for the development of preventive and therapeutic strategies against mastitis [53,54,55,56]. Recently, a whole milk stimulation assay was established for evaluating the response of human milk immune cells to in vitro stimulation with pathogens [37]. The advantage of this method relies on preserving the microenvironment of interaction between the pathogen and milk immune cells as it presents in vivo. In the present study, in vitro stimulation of whole camel milk was performed to investigate the impact of some TLR ligands on the phenotype and function of mammary gland immune cells.

Pathogen sensing by the innate sentinel cells is critical for early immune mediator production and triggering the inflammatory response [12]. The activation of TLRs by their interaction with the pathogen-associated molecular patterns (PAMPs) represents the first step of the inflammatory reaction [57]. Lipopolysaccharide (LPS), which is a PAMP associated with acute mammary gland infections with gram-negative bacteria [18,19], stimulates the innate immune system through TLR4 in cooperation with the surface molecule CD14, the serum LPS-binding protein, and the MD-2 adaptor protein, leading to the production of several inflammatory mediators [20,21,22,58]. On the other hand, Pam3CSK4 is a PAMP that activates TLR1/2 representing innate immune responses to gram-positive bacteria [23,24,59]. In the bovine udder, the expression of TLR2 and TLR4 was described in several immunostaining studies [60,61]. This is also supported by functional studies showing the modulatory effects of the intramammary infusion with the bacterial PAMPs that stimulate TLR2 or TLR4 ligands [1,18]. Due to the lack of monoclonal antibodies to camel TLR, we investigated their functional expression by the analysis of TLR-agonist-induced responses in milk immune cells. The TLR activation leads to essential processes in innate immune cells, including the generation of ROS, degranulation, NET formation (NETosis), and cytokine production [62]. In the present study, the observed change in the granulocytes’ side scatter properties, which correlates with cell granularity [63], indicates the potential of the two TLR ligands, LPS and Pam3CSK4, to induce activation and degranulation of milk immune cells. This is also supported by the observed change in the expression of the cell surface molecules MHCII [64] and CD18 [65], which are activation markers of macrophages and neutrophils, respectively. Although it has not been proven using immunostaining with TLR-specific antibodies, the activation of several cellular activities in LPS- or Pam3CSK4-stimulated milk indicates the expression of TLR4, TLR1, and TLR2 on camel milk cells.

After ingesting and killing pathogens, neutrophils undergo constitutive programmed cell death, a process that is essential in the resolution of inflammation [66]. In the present study, although the number of viable cells (the fraction of cells with negative PI staining) separated from stimulated whole milk did not differ significantly from the values measured for the cells separated from the collected milk samples before incubation (data not shown), stimulation with either LPS, Pam3CSK4, or PMA resulted in a higher proportion of apoptotic milk cells. Neutrophil apoptosis has been linked to an increased abundance of the hyaluronic acid receptor CD44, which mediates their recognition and elimination by macrophages, preventing the tissue damage caused by the release of neutrophil constituents [67,68,69]. A regulatory role for CD44 has been therefore, described in several inflammatory responses triggered by TLR signaling, leading to resolving the inflammation [70,71]. In the present study, the upregulation of CD44 on milk cells, although not proved specifically for separated milk neutrophils, may be considered a regulatory mechanism of the inflammatory response in the camel mammary gland by mediating the elimination of apoptotic neutrophils by macrophages. While PMA induces apoptosis through the activation of serine proteases and caspase-3/CPP32 [72], the effect of TLR-agonists on cell apoptosis and the molecular mechanisms through which TLR ligation may regulate apoptosis are very complex [73]. For the TLR ligands, LPS and Pam3CS4K, pro- as well as anti-apoptotic effects have been described depending on the cell type [74,75]. The different pro-apoptotic potentials of PMA and the TLR agonists LPS and Pam3CS4K on milk cells could be due to the different signaling pathways involved [76] or could be a result of the pro- and anti-apoptotic effects of the TLR agonists on the different immune cell types in the milk. Therefore, future research should assess the cell type-specific effects of TLR stimulation on the vitality of sorted milk cells.

Phagocytosis, ROS production, and NET-formation are key antimicrobial functions of milk phagocytes [77,78,79]. An enhancing effect of TLR-signaling on the bactericidal function of macrophages has been reported previously. The activation of surface TLR4 or TLR2/1 through their ligands LPS and Pam3CSK4, respectively, results in the recruitment of mitochondria to macrophage phagosomes and the augmentation of ROS production in a TNF receptor-associated factor 6-mediated process [80,81]. In the current study, stimulation with LPS or Pam3CSK4 did not affect the phagocytosis activity of the milk cells, which contrasts with the observed phagocytosis-enhancing effect of PMA stimulation. On the other hand, only stimulation with Pam3CSK4 induced ROS production in the milk cells, which was comparable with the PMA-induced response. Promoting effect of LPS and Pam3CSK4 on the antimicrobial activity of neutrophils (phagocytosis, respiratory burst, adhesion molecules expression, cytokine production) has been described for highly purified human neutrophils [82,83]. Our results are in line with a previous study reporting a selective ROS-inducing effect for Pam3CSK4 but not for LPS on bovine neutrophils [84]. In addition, the lack of a modulating effect for LPS and Pam3CSK4 on the phagocytosis activity of milk phagocytes may be due to the complex cellular composition of the milk sample. Therefore, further studies are required using purified cell populations to investigate the cell-type-specific immunomodulating properties of the TLR ligands.

In the present study, although both LPS and Pam3CSK4 stimulated NETosis in camel milk cells, the stimulating effect of Pam3CSK4 was significantly higher than LPS and was comparable to that of PMA. The role of different TLRs in NETosis was recently investigated for human neutrophils [85,86]. Although the mechanisms behind the TLR-induced NET formation are still not fully understood, a role for ROS production in NET formation was recently reported [87,88]. In the present study, the difference in ROS production observed after stimulation with LPS and Pam3CSK4 may have contributed to the difference in the NETosis potential of those TLR ligands.

In vitro stimulation of bovine mammary gland epithelial cells with LPS and Pam3CSK4 differentially modulates the gene expression of several cytokines and chemokines. In contrast to stimulation with LPS, which resulted in the significant upregulation of the chemokines CCL2, CXCL2, CXCL3, and CXCL8, and the inflammatory cytokine IL1, Pam3CSK4 did not modulate the expression of the cytokines or chemokines [89]. In contrast to the bovine system, the observed higher ROS and NETosis response of the camel milk phagocytes to stimulation with Pam3CSK4, compared to stimulation with LPS, indicates the higher potential of Pam3CSK4 than LPS to stimulate inflammatory responses in the camel udder. Future work should investigate whether different TLR signaling and molecular mechanisms exist in camel and cattle mammary glands.

Although the stimulants LPS and Pam3CS4K were selected as PAMPs that are representative of gram-negative and gram-positive bacteria, respectively, these molecules will not completely reflect real bacterial mastitis. Therefore, further studies with experimental infection of the mammary gland with live bacterial pathogens are required for the characterization of the in vivo immune response of the camel mammary gland to mastitis pathogens.

## 5. Conclusions

In conclusion, we demonstrated different modulating effects of the TLR ligands LPS and Pam3CSK4 on selected functions of camel milk immune cells. Our results may have implications for the use of synthetic TLR agonists as immunomodulatory adjuvants of the immune response to intra-mammary vaccines against mastitis pathogens. Further studies are required for a comprehensive understanding of the immune response of the camel mammary gland to specific pathogens which would contribute to the development of effective mastitis control strategies.

## Data Availability

The datasets used and/or analyzed during the current study are available from the corresponding author on reasonable request.
